# Emerging Inter-Swarm Collaboration for Surveillance Using Pheromones and Evolutionary Techniques

**DOI:** 10.3390/s20092566

**Published:** 2020-04-30

**Authors:** Daniel H. Stolfi, Matthias R. Brust, Grégoire Danoy, Pascal Bouvry

**Affiliations:** 1 SnT, University of Luxembourg, L-4364 Esch-sur-Alzette, Luxembourg; matthias.brust@uni.lu (M.R.B.); gregoire.danoy@uni.lu (G.D.); pascal.bouvry@uni.lu (P.B.); 2 FSTM/DCS, University of Luxembourg, L-4364 Esch-sur-Alzette, Luxembourg

**Keywords:** swarm robotics, mobility model, inter-swarm collaboration, unmanned aerial vehicle, unmanned ground vehicle, evolutionary algorithm, pheromones, bio-inspiration

## Abstract

In this article, we propose a new mobility model, called Attractor Based Inter-Swarm collaborationS (ABISS), for improving the surveillance of restricted areas performed by unmanned autonomous vehicles. This approach uses different types of vehicles which explore an area of interest following unpredictable trajectories based on chaotic solutions of dynamic systems. Collaborations between vehicles are meant to cover some regions of the area which are unreachable by members of one swarm, e.g., unmanned ground vehicles on water surface, by using members of another swarm, e.g., unmanned aerial vehicles. Experimental results demonstrate that collaboration is not only possible but also emerges as part of the configurations calculated by a specially designed and parameterised evolutionary algorithm. Experiments were conducted on 12 different case studies including 30 scenarios each, observing an improvement in the total covered area up to 11%, when comparing ABISS with a non-collaborative approach.

## 1. Introduction

Unmanned Aerial Vehicles (UAV), also known as “drones”, have become commonplace devices in the 21st century. Their use ranges from toys to military devices, while their cost can vary from tens to millions of dollars. For example, the word drones appeared in more than 500 articles in the New York Times from January 2012 to May 2016 [1] and the number of published papers has increased from 544 in 2013 to more than 1593 in 2017 [2]. Nowadays, these vehicles present several advantages for the society even though there are many concerns about privacy and civil liberties [3].

Although drones are frequently related with defence activities and projects [4], unarmed drones may also be strategically relevant due to their great surveillance capabilities [5]. In fact, they are also used in many other activities such as cargo delivery [6], road traffic surveillance [7], fire fighting [8], and to herd a flock of birds away from an airport [9].

Besides UAVs (fixed wing or multi-rotors), there exists other types of unmanned autonomous vehicles, such as Unmanned Ground Vehicles (UGV). Each vehicle type has its own characteristics, e.g., the type of areas they are able to explore. For instance, UAVs are able to move freely in the air and fly over water surfaces, while UGVs can explore dense forest areas where UAVs are unable to adventure. Using one single device in such scenarios is not an option. Where the collective intelligence emerges is in homogeneous swarms composed of several unmanned vehicles moving in an autonomous and coordinated manner in the air, on the ground and in the sea, following a bio-inspired approach.

Animal collective motion can be observed in a wide range of biological systems, such as bacteria colonies, fish schools and bird flocks [10]. In many cases, a swarm of individuals is able to solve a complex problem which is intractable for a single individual. This swarm intelligence is frequently seen in research works involving robots as it increases the robustness of a system and facilitates the cooperation among individuals in the swarm [11].

One well-known way of collaboration is found in evolutionary game theory (EGT) where individuals compete and sometimes collaborate with each other following evolutionary strategies [12]. This behaviour, consisting in achieving a common good while seeking a personal reward, has been applied to solve several problems such as predicting infectious diseases with vaccination strategies [13], making military decisions [14], optimising packet relaying in wireless networks based on reciprocity [15] and, now, improving surveillance missions sharing pheromone trails.

Our proposal consists of a new mobility model, called ABISS, for homogeneous multi-swarms of autonomous unmanned vehicles of different types. By using ABISS, collaborations between swarms emerge after applying evolutionary bio-inspired techniques, with the common objective of improving area coverage while performing surveillance. This is a distributed intelligence system, in which, if some members fail, the rest of the swarm still carries on their surveillance task, increasing the robustness of the proposal.

The main contributions of this paper are:

1. A new mobility model (ABISS) is designed to be used by unmanned vehicle swarms as a cooperation strategy for mutual inter-swarm collaboration.
2. An Evolutionary Algorithm (EA) is specially designed and tuned for optimising the ABISS’ parameters to maximise the coverage of the area under surveillance.

The rest of this paper is organised as follows. In the next section, we review the state of the art related to our work. In Section 3, our proposal is discussed. The problem definition and its instances are described in Section 4. Section 5 presents our optimisation algorithm and its hyper-parameterisation. The discussion of the results is in Section 6. Finally, in Section 7, conclusions and future work are given.

## 2. Related Work

Several articles focus on swarm robotic problems [16,17]; we review some of them related to our bio-inspired approach. Some articles use pheromone-based models to control the movement of robots and vehicles. In [18], the authors introduced a collective autonomous system inspired by the thermotactic aggregation behaviour of honeybees. They studied this probabilistic system at a mesoscopic scale by limiting the sensing of their robots and observed a collective decision-making behaviour when locating energy sources. A coordination model using indirect communication performed by repulsive pheromone interactions is proposed in [19]. In that article, five strategies related to the next-cell selection are evaluated according to the performance of surveillance tasks. The proposed model is based on a two-dimensional Cellular Automata and the two testing environments are composed by seven and ten rooms, respectively. In [20], the authors proposed a bio-inspired algorithm which allows communication between robots using pheromones as collective memory. They tested their proposal in square scenarios made of 25 × 25 and 50 × 50 cells and reported it to be better than the other algorithms included in the comparison. A surveillance system using an environment virtually partitioned into balanced disjoint areas is presented in [21]. One robot capable of releasing and distinguishing its own pheromones from that of others is assigned to each area and restricted to it. The authors tested their proposal in two scenarios divided into rooms and identified two phases: dispersion and navigation confinement.

Other articles use evolutionary techniques and swarm intelligence algorithms applied to robots to achieve their goals. In [22], the authors used evolutionary robotics to synthesise a collegial decision making behaviour similar to the one displayed by cockroaches when selecting a resting shelter. They experimented using ARGoS [23], a simulator of swarm robotics systems, using an Artificial Neural Network (ANN) as controller and an EA for setting up its parameters. In [24], a combination of novelty search (an evolutionary process based on the promotion of phenotypic diversity) and NEAT (NeuroEvolution of Augmenting Topologies) is used to evolve neural controllers for homogeneous swarms of robots. The authors conducted their study in a simulator where the robots performed tasks of aggregation and sharing a recharging station.

A Self-aware Communication Strategy (SCS) for controlling the heading direction of a swarm of robots is proposed in [25]. The authors implemented a small group of informed agents which have to guide a large swarm in a stationary environment having different number of goals. They experimented with simulated and real robots to evaluate the ability of the swarm to follow the leaders when changing direction as well as the swarm’s cohesion. In [26], a Genetic Algorithm (GA) is proposed to optimise the parameters of a swarm of robots in order to improve the foraging of dispersed resources and mapping the environment. Those parameters define how ants, simulated by Agent-Based Models (ABM), travel from their nest, search and use site fidelity and pheromone communication. Then, the ABM was modified to model the swarm of robots, which was used in the simulations performed to show that pheromone-like communication improved foraging success.

In [27], a realistic simulation framework is presented and two decentralised control algorithms are applied to a group of autonomous quadcopters in a three-dimensional space. One is based on a simple self-propelled flocking and the other is a collective target tracking algorithm. By conducting a series of experiments, the authors showed the applicability of their proposal and its stability when using a repulsion force to avoid collisions and an alignment force to align the heading directions of neighbours. In [28], the authors studied two informative path planning problems motivated by the use of robots in precision agriculture. They introduced a new planning problem, which penalises the time spent in travelling and the time spent for obtaining measurements. UAVs were used to maximise the number of visited points, and, since they had limited autonomy, they could land on the only UGV in the system, to be carried between different points without expending battery.

These studies propose mobility strategies which do not include collaborations between swarms of different types of unmanned autonomous vehicles. Some of them use evolutionary robotics or communication using pheromones as we do. However, these articles use a different mobility model and the surveillance scenarios are different from the ones proposed in this paper. Our proposal focuses on the communication and collaboration between different swarms of vehicles using pheromones and attractors. It relies on a distributed intra-swarm mobility model [29], which generates unpredictable trajectories using a chaotic solution of a dynamical system [30], combined with pheromone repel mobility [31]. The new model we propose is fully parameterised to achieve better coverage results and these new parameters are calculated by using an EA, specially adapted to optimise the covered area and foster inter-swarm collaboration. This approach plus the combination of inter-swarm and intra-swarm mobility, to the best of our knowledge, has not been used before.

## 3. Attractor Based Inter-Swarm CollaborationS (ABISS)

**Figure 1:**
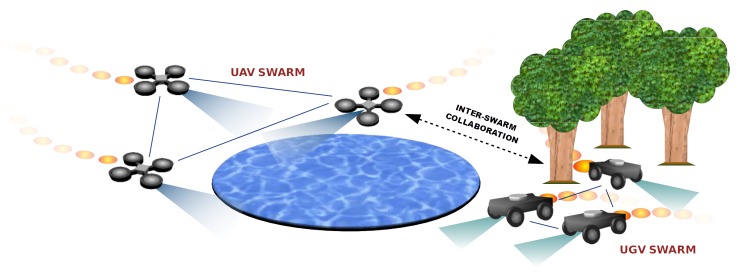
Attractor Based Inter-Swarm collaborationS (ABISS). In this example, a UAV swarm explores a water surface that UGVs cannot access.

In this article, we present a new mobility model called ABISS (Attractor Based Inter-Swarm collaborationS), to be used by swarms of unmanned vehicles, e.g., Unmanned Aerial Vehicles (UAV) and Unmanned Ground Vehicles (UGV). Having different swarms of robots allows exploring all the area under surveillance taking advantage of each swarm’s characteristics. For example, UAVs are able to watch lakes and ponds while UGVs can do it efficiently in dense forests (Figure 1). On the contrary, UGVs cannot access water surfaces and UAVs have many troubles flying among trees. We call these areas *unreachable zones* (UZ) by a given type of vehicle.

Our proposal aims to compensate the limitations of each swarm complementing the unpredictable trajectories generated at intra-swarm level with inter-swarm collaboration strategies using evolutionary techniques. These two main components of ABISS are used to efficiently cover all the area under surveillance.

### 3.1. Intra-Swarm Mobility

Each swarm is homogeneous, consisting of the same type of vehicles, which explore the area under surveillance following unpredictable trajectories. Since unpredictability is needed for avoiding interception, the CACOC (Chaotic Ant Colony Optimisation for Coverage) algorithm [29] is the foundation of our intra-swarm mobility model.

CACOC combines a Chaotic Rössler Mobility Model (CROMM) with pheromone repel mobility [31] to obtain an intra-swarm mobility model in which each vehicle chooses a direction depending on the pheromone trail that surrounds it. Chaotic mobility generates routes that are unpredictable for an external observer although the vehicles’ trajectories are still deterministic since they depend on the initial conditions of the chaotic system. This is extremely valuable in case there are telemetry issues, allowing the Ground Control Station (GCS) to predict where the vehicles are until communications are re-established. This is possible since the GCS (and only the GCS) knows the initial conditions currently selected for the mobility model. Different trajectories can be obtained by selecting other different initial conditions for the chaotic attractor (we called them scenarios of a case study, see Section 4.2). Those trajectories are still predictable for the GCS.

Algorithm 1 shows the pseudocode of CACOC where after setting the default current state the main loop begins. In this loop, the first return map ρ describing a chaotic system (chaotic attractor obtained by solving a system of differential equations, see [32,33]) is used to replace the random part of the mobility model, as it was proven to perform better [29]. If there are no pheromones in the neighbourhood, CROMM is used. CROMM is a purely chaotic model which decides the next move using an equally divided partition, so that if the first returned map value ρ is less than $\frac{1}{3}$, the vehicle will move to the right; if ρ is between $\frac{1}{3}$ and $\frac{2}{3}$, the vehicle will move to the left; and it will move ahead otherwise [29].

If pheromones are detected, the amount of them on the right ($P_{R}$) and left ($P_{L}$) are calculated and used to decide the next movement direction, as shown in Table 1. The idea is moving away from the higher amount of pheromones detected [29], taking into account the value of ρ as a probability. By doing so, the vehicles tend to avoid revisiting the same area in a short period of time, exploring more interesting regions until it is time to come back (when the pheromones have evaporated) and recheck the places already visited. We improved CACOC as intra-swarm mobility model [34,35] and integrated it into ABISS by optimising its parameters to work with the inter-swarm mobility model.
**Algorithm 1** Pseudocode of CACOC. **procedure**
CACOC  currentstate←“ahead”  **loop**   ρ←next value in first return map   **if**
no pheromone sensed in the neighbourhood
**then**    currentstate←CROMM   **else**    **if**
$\rho <P_{R}$
**then**     currentstate←“right”    **else if**
$\rho <P_{R}+P_{L}$
**then**     currentstate←“left”    **else**     currentstate←“ahead”    **end if**    move according to the currentstate   **end if**  **end loop** **end procedure**

### 3.2. Inter-Swarm Mobility

In our proposal, vehicles of different types evolve in homogeneous swarms which interact with each other having a common objective of maximising the covered area (Figure 1). In scenarios where every vehicle can perform its duty without finding unreachable areas, the collaboration is given by the pheromone repel model (CACOC). However, when there are unreachable areas, a new extra virtual element is used to foster the collaboration between swarms. We called it *attractor*.

Attractors are specific for a given type of vehicles and are left behind when a vehicle finds an unreachable area, i.e., a UGV finds a water surface and leaves an attractor for UAVs. Following this example, when a UAV detects this attractor, it might decide to ignore it or, in turn, ignore the possible pheromone trails and head to the attractor. This decision also depends on the probabilistic model based on chaotic dynamic systems and the collaboration probability of the model. Since collaboration implies changing the vehicle’s original trajectory, which might sacrifice the global coverage, improving the local exploration of unreachable zones is seen by each vehicle as a reward. The collaboration willingness as well as the rest of parameters of the model are calculated and optimised by the proposed EA, described in Section 5.1.

**Figure 2:**
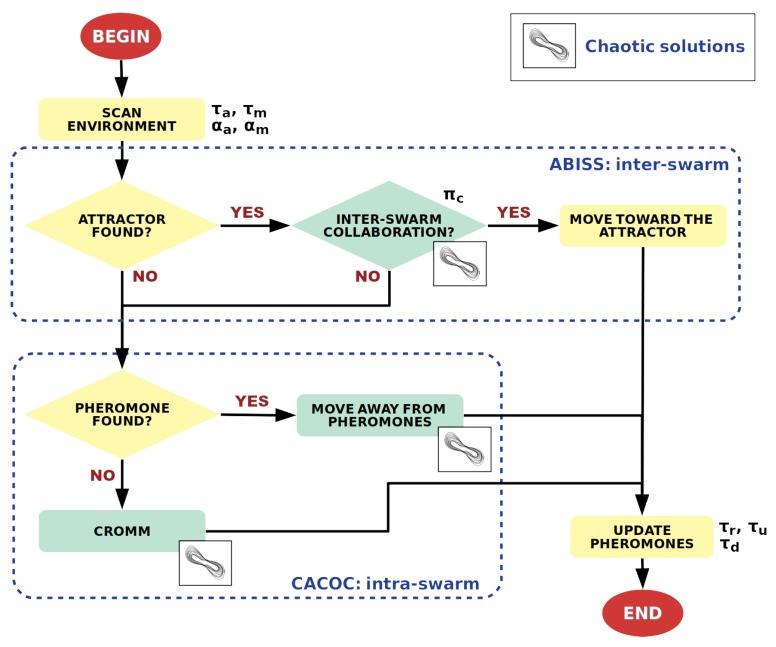
Flow diagram of ABISS using attractors, chaotic solutions and pheromones. The parameters affecting each operation are listed next to them.

The flow diagram of ABISS is shown in Figure 2. It is executed by each vehicle being part of this distributed system. At each step, a vehicle scans the environment in front of it looking for an attractor for its own type. If it finds one, a possible collaboration is taken into account according to the vehicle’s collaboration probability and the next value in the sequence of chaotic solutions, previously calculated (offline). If the vehicle decides to collaborate, it will move toward the attractor. If not, any pheromone trail found during the scanning will be taken into account to decide the next moving direction (intra-swarm). Attractors work as the opposite to pheromones which are repellents. While pheromones evaporate over time, attractors only disappear when a vehicle reaches them. The new parameter set used to modify the vehicles’ behaviour and efficiency is described in the next section.

### 3.3. Model Parameters

**Figure 3:**
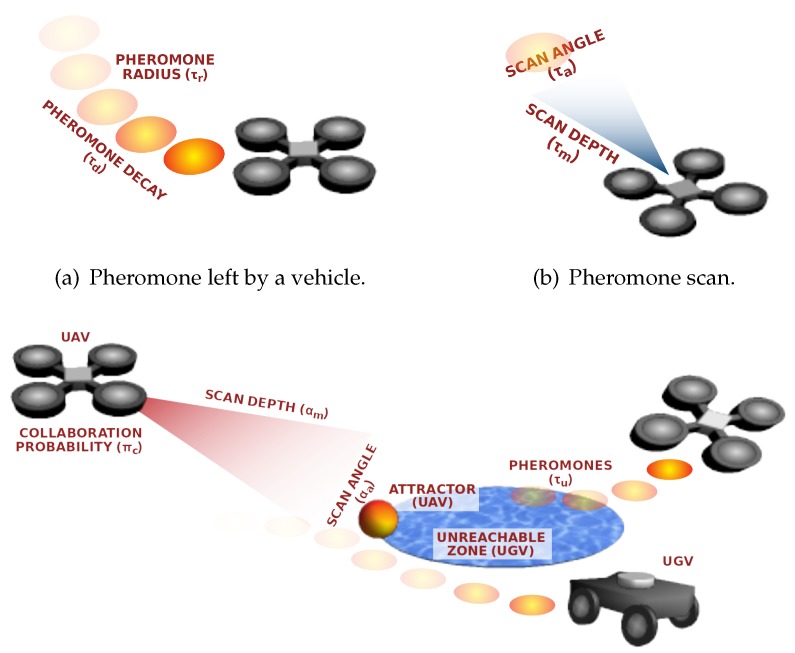
Parameters of ABISS. The UAV has to decide whether to go to explore the water surface where the UGV has left an attractor because it could not enter.

We have defined a set of parameters to control how the vehicles move and how they react and collaborate. Since pheromones modify the vehicles’ movement, we have parameterised the percentage of pheromone evaporation ($\tau_{d}$) at each step (one second in our study) and the amount of pheromones left by each vehicle ($\tau_{r}$), as depicted in Figure 3a). Since vehicles are able to detect pheromones by scanning the area they have ahead, we have set two other parameters, namely scanning angle ($\tau_{a}$) and maximum detection distance ($\tau_{m}$), as shown in Figure 3b.

Additionally, four other parameters are used when there exist UZs in the area and the inter-swarm collaboration is possible. Concretely, when a vehicle is diverted because it was unable to enter a zone, e.g., a water surface, it leaves an attractor to require collaboration to members of other swarms. Then, when a vehicle in another swarm detects the attractor by scanning the area with an angle ($\alpha_{a}$) and depth ($\alpha_{m}$), it has to decide whether it is willing to collaborate or not, according to the collaboration probability ($\pi_{c}$), as shown in Figure 3c. The pheromones left in UZs (when a vehicle is exploring them) are also subject to a decay rate ($\tau_{d}$), and the initial amount left by a vehicle is controlled by $\tau_{u}$.

Table 2 shows the parameters of our mobility model to be optimised by the proposed EA, in order to maximise the covered area. If we look at the third column, we can see that there are different types of parameters, which implies having a heterogeneous solution vector. When there are no unreachable zones, the solution vector consists of the first four parameters in Table 2, i.e., $\vec{x}=\left(\tau_{d},\tau_{r},\tau_{a},\tau_{m}\right)$. These are the new parameters we have defined for the intra-swarm model. When we optimise the more complex scenarios having unreachable zones, the solution vector contains all the parameters, including now the inter-swarm model, i.e., $\vec{x}=\left(\tau_{d},\tau_{r},\tau_{a},\tau_{m},\pi_{c},\alpha_{a},\alpha_{m},\tau_{u}\right)$.

## 4. Problem Description

The problem we are addressing in this paper is the intelligent surveillance of a geographical area to detect possible intruders by maximising the area covered by a multi-swarm of vehicles. The next sections describe the objective function to be maximised, the problem instances used to test our proposal and the parameter sensitivity analysis.

### 4.1. Objective

In our model, the vehicles move by an environment composed by a lattice of 1 × 1-m${}^{2}$ cells, following the rules of ABISS. Previous studies [36] have validated this cellular approach as a good representation of real scenarios involving actual drones. Since each vehicle has its own camera, we calculate the number of cells explored according to the detection zone of each vehicle (a 3 × 3-cell square centred on the vehicle). Having this in mind, the objective function consists in the maximisation of the number of explored cells with respect to the total amount of cells in the scenario. After configuring the vehicles, we evaluate each configuration as the percentage of the area visited during the simulation time (600 s). Concretely, for each case study (see Section 4.2), we calculate its fitness value (phenotype) for a vehicle configuration (genotype) given, as shown in Equation (1).

(1) $$F\left(\vec{x}\right)=\frac{1}{\eta }\sum_{i=1}^{\eta }\frac{\#ofexploredcells{\left(\vec{x}\right)}_{i}}{\#ofcellsinthescenario_{i}},\;\eta =\#ofscenarios$$

The fitness function *F* is calculated for the solution vector $\vec{x}$, by simulating η scenarios (30 in our case) to obtain the number of explored cells to be divided by the total number of cells in the scenario (it calculates the proportion of explored cells in each scenario *i*). We evaluate 30 different scenarios of each case study using the Monte Carlo method [37] to increase the robustness of the configuration found after the optimisation process. As we are maximising the explored area, the higher is the value of $F(\vec{x})$, the better.

### 4.2. Instances

We generated four map layouts to evaluate ABISS and compare it with the parameterised CACOC (our approach using inter-swarm collaborations vs. inter-swarm only mobility). The first layout does not include unreachable zones (UZ) and the other three have one zone of dense forest unreachable by UAVs and another zone consisting of a water surface unreachable by UGVs. These UZ areas represent 18% of the total area available in the maps. Furthermore, we wanted to test our proposal on two different map sizes, as well as use different numbers of vehicles to address several possible swarm configurations. Hence, we defined the 12 case studies shown in Table 3. The nomenclature used consists of the map dimensions and the number of vehicles, followed by the letter *z* if there are UZs in the map. Finally, the number next to *z* corresponds to the map’s layout.

As explained above, the trajectories followed by the vehicles depend on the chaotic solutions of a dynamical system. Consequently, these solutions are sensitive to the initial conditions. To take advantage of this characteristic, we defined 30 different scenarios (instances) of each case study (using 30 different initial conditions for the chaotic attractor), in which the vehicles will move using different trajectories. As a result, the covered area in each scenario is expected to be also different.

**Figure 4:**
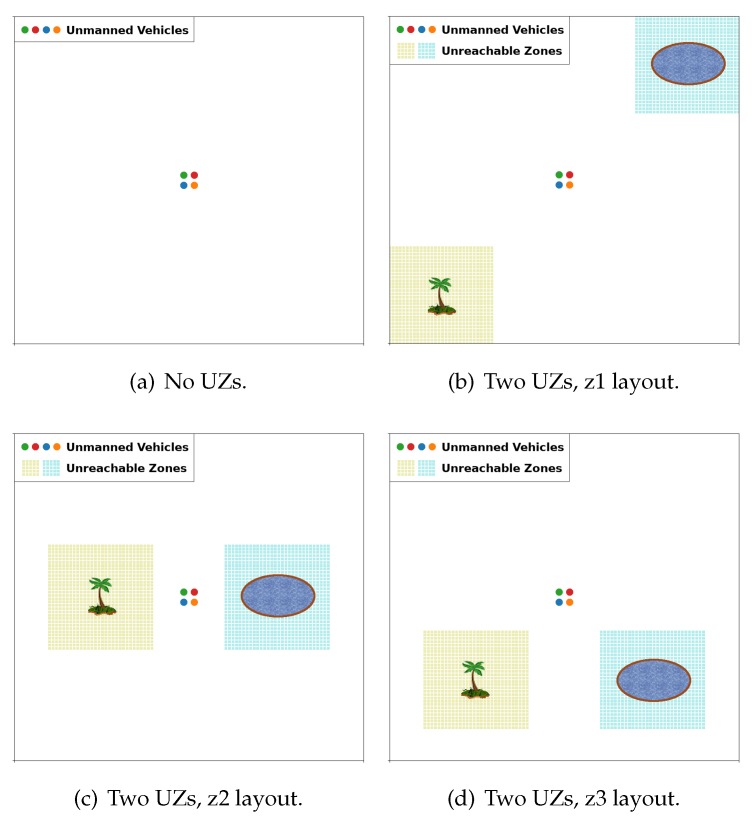
Case studies without (**a**) and with square unreachable zones (**b**–**d**). In these examples, there are four vehicles placed at their starting position in the centre of the map.

Figure 4a shows a full accessible map (100x100.4), while Figure 4b (100x100.4z1), Figure 4c (100x100.4z2), and Figure 4d (100x100.4z3) are maps having two unreachable zones, one by UAVs (green) and the other by UGVs (cyan).

### 4.3. Parameter Sensitivity Analysis

We performed a sensitivity analysis to identify which parameters (factors) are the most important in our novel model of intra-swarm mobility and inter-swarm collaboration when we measure the total area covered by vehicles.

We used the method proposed by Morris [38] which is based on computing incremental ratios for each system’s parameter that are then averaged to determine its importance. This method analyses the distribution of values by using trajectories of measurement points in the parameters’ space. As a result, the mean and standard deviation σ of the factor distribution are given. In our study, we used the revised mean called $\mu^{★}$ (the estimate of the mean of the distribution of the absolute values of the elementary effects), which can be used by itself to provide a reliable ranking [39]. Morris’ method presents low computation cost compared to others, especially in systems having high number of parameters as the one analysed here.

**Figure 5:**
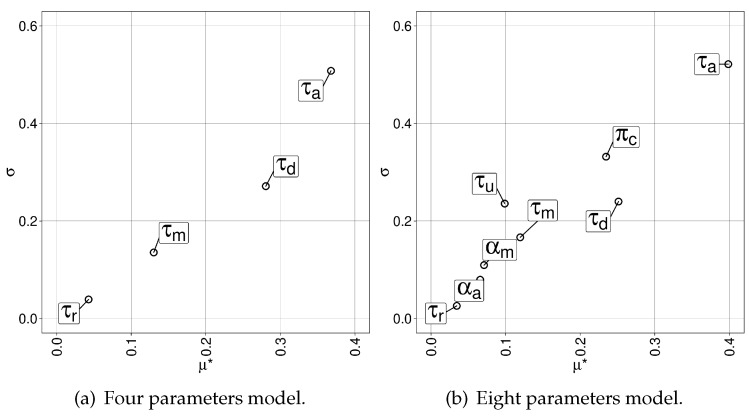
Parameter sensitivity study (σ and $\mu^{★}$) for the model using four and eight parameters to modify the behaviour and performance of ABISS.

After the evaluation of 100 configurations of the model having four parameters (intra-swarm) and 180 of the one having eight parameters (intra-swarm plus inter-swarm), we obtained the values of σ and $\mu^{★}$ for each parameter, shown in Figure 5a,b, respectively. Note that each evaluation was performed on the 30 existing instances of 100x100.6z1, which amounts to 8400 evaluations in total.

It can be seen that the pheromone scan angle ($\tau_{a}$) and its evaporation rate ($\tau_{d}$) have the biggest influence over the system. Then, when collaborations are possible, its probability ($\pi_{c}$) is also reported as an important parameter, as expected. Surprisingly, the scan depth and angle ($\tau_{m}$ and $\alpha_{m}$) are less decisive, and the pheromone radius ($\tau_{r}$) turned out to have the least effect over our mobility model.

## 5. Optimisation Approach

In this section, we propose an Evolutionary Algorithm (EA) to optimise the parameters of ABISS to maximise the area explored by vehicles. After that, we address the parameterisation of our EA using an automatic method for algorithm configuration (irace).

### 5.1. Evolutionary Algorithm (EA)

Our EA is based on a Genetic Algorithm (GA) [40,41], which is an efficient method for solving combinatorial optimisation problems. Genetic Algorithms simulate processes present in evolution such as natural selection, gene recombination after reproduction, gene mutation and the dominance of the fittest individuals over the weaker ones. A typical GA consists of a population of μ individuals from which a subset is selected using a selection operator. Then, these individuals are recombined using a crossover operator to obtain a new set of λ individuals based on the original population. After that, a probabilistic mutation is applied introducing little modifications to the individuals’ chromosome. Finally, after evaluating the new offspring, the replacement operator selects the fittest to replace the former population. We have chosen an evolutionary bio-inspired approach for solving this difficult real problem which requires high evaluation times and has a huge search space that is very difficult to explore using exhaustive methods. Additionally, there is no analytic equation, thus traditional methods are not viable. Moreover, since we work with a population of individuals (instead of one solution at a time), the evaluation of each generation can be easily parallelised, noticeably reducing execution times. All these reasons as well as its low complexity operations make EA one of the best options for exploring the search space and solving our optimisation problem [42].

The pseudocode of our EA is shown in Algorithm 2. We designed a generational EA where λ=μ, i.e., the auxiliary population *Q* contains the same number of individuals as the population *P*. The parameters of EA (to be optimised in Section 5.2) are the number of individuals $N_{i}$, the crossover probability $P_{c}$ and the mutation probability $P_{m}$.

First, after initialising *t* and Q(0), P(0) is generated by the *Initialisation* function. To increase the diversity of P(0), we generated 100μ individuals and selected the μ most different ones according to their entropy. Then, the main loop is executed while the *TerminationCondition* is not fulfilled (in our case, we stop after 100,000 evaluations). Inside the main loop, the *Selection* operator is applied to fill the working population Q(t) using Binary Tournament [43]. Next, the *Crossover* operator is applied and, after that, the *Mutation* operator modifies the new offspring slightly. These two operators, based on the Continuous Genetic Algorithm (CGA) presented in [44], are discussed below. Finally, after the *Evaluation* of Q(t) using the evaluation function (Equation (1)), the new population P(t+1) is obtained by applying the *Replacement* operator. To avoid population stagnation and preserve its diversity, instead of using elitism [40], we selected the best individual in Q(t) to replace the worst one in P(t) if it is better (it covers a bigger area), as proposed in [44].
**Algorithm 2** Pseudocode of the Evolutionary Algorithm (EA). **procedure**
EA($N_{i},P_{c},P_{m}$)  t←∅  Q(0)←∅           ▹ Q = auxiliary population  $P\left(0\right)\leftarrow Initialisation\left(N_{i}\right)$      ▹ P=population  **while**
notTerminationCondition()
**do**   Q(t)←Selection(P(t))   $Q\left(t\right)\leftarrow Crossover(Q\left(t\right),P_{c})$   $Q\left(t\right)\leftarrow Mutation(Q\left(t\right),P_{m})$   Evaluation(Q(t))   P(t+1)←Replacement(Q(t),P(t))   t←t+1  **end while** **end procedure**

#### 5.1.1. Crossover Operator

The pseudocode of the crossover operator is shown in Algorithm 3. It is based on the one presented in [44]. However, we adapted it to our heterogeneous solution vector so that it can work not only with real values but also with integer ones.
**Algorithm 3** Pseudocode of the crossover operator. **function**
Crossover($Q,P_{c}$)  $Q^{\prime }\leftarrow \emptyset$  **for all**
$\{\vec{x},\vec{y}\}\in Q$
**do**           ▹ all the individuals in *Q*, taken in pairs   ${\vec{x}}^{\prime }=\vec{x}$   ${\vec{y}}^{\prime }=\vec{y}$   **if**
$rnd\left(\right)<P_{c}$
**then**                    ▹ crossover probability    p←randInt(1,L)    ▹ crossing point *p*, *L* is the length of the solution vector    **for**
i←p,L
**do**                      ▹*i* goes from *p* to *L*     **if**
is_an_int_parameter(i)**then**        ▹ type of the *i*-th parameter      ${\vec{x}}^{\prime }\left[i\right]=\vec{y}\left[i\right]$      ${\vec{y}}^{\prime }\left[i\right]=\vec{x}\left[i\right]$     **else**      M←randInt(1,10)      $\Delta_{x}\leftarrow \vec{x}\left[i\right]/M$      $\Delta_{y}\leftarrow \vec{y}\left[i\right]/M$      ${\vec{x}}^{\prime }\left[i\right]=\vec{x}\left[i\right]+\Delta_{y}-\Delta_{x}$      ${\vec{y}}^{\prime }\left[i\right]=\vec{y}\left[i\right]-\Delta_{y}+\Delta_{x}$     **end if**    **end for**   **end if**   $Q^{\prime }\leftarrow Q^{\prime }\cup \left\{{\vec{x}}^{\prime },{\vec{y}}^{\prime }\right\}$  **end for**  **return**
$Q^{\prime }$ **end function**

First, two individuals $\vec{x}$ and $\vec{y}$ are taken from the population *Q* and copied to the destination ${\vec{x}}^{\prime }$ and ${\vec{y}}^{\prime }$. If a generated random number is less than the crossover probability $P_{c}$, the recombination process begins by selecting the crossing point using another random integer value (*p*), generated between one and the length of the solution vector *L* (four or eight). If the current position (*i*) corresponds to an integer parameter (it only takes integer values), the vectors’ values in position *i* are swapped. On the contrary, if it is a real-value parameter, the vectors’ values are modified according to another random integer value (*M*) by calculating $\Delta_{x}$ and $\Delta_{y}$, as shown in Algorithm 3. This process is repeated for the rest of the individuals in the working population *Q* (taken in pairs) to obtain the new population $Q^{\prime }$.

#### 5.1.2. Mutation Operator

The pseudocode of the mutation operator is shown in Algorithm 4. It is also based on the idea presented in [44] but in our implementation it has been adapted to our problem characteristics.
**Algorithm 4** Pseudocode of the mutation operator. **function**
Mutation($Q,P_{m}$)  $Q^{\prime }\leftarrow \emptyset$  **for all**
$\{\vec{x}\}\in Q$**do**             ▹ all the individuals in *Q*   ${\vec{x}}^{\prime }\leftarrow \vec{x}$   **for**
i←1,L
**do**  ▹*i* goes from 1 to *L* (the length of the solution vector)    **if**
$rnd\left(\right)<P_{m}$
**then**            ▹ mutation probability     M←randInt(1,10)     $\Delta \leftarrow getDelta(\vec{x}\left[i\right],M)$     **if**
rnd()<0.5
**then**           ▹ increment/decrement      Δ=−1∗Δ     **end if**     ${\vec{x}}^{\prime }\left[i\right]\leftarrow \vec{x}\left[i\right]+\Delta$    **end if**   **end for**   $Q^{\prime }\leftarrow Q^{\prime }\cup \left\{{\vec{x}}^{\prime }\right\}$  **end for**  **return**
$Q^{\prime }$ **end function**

**Figure 6:**
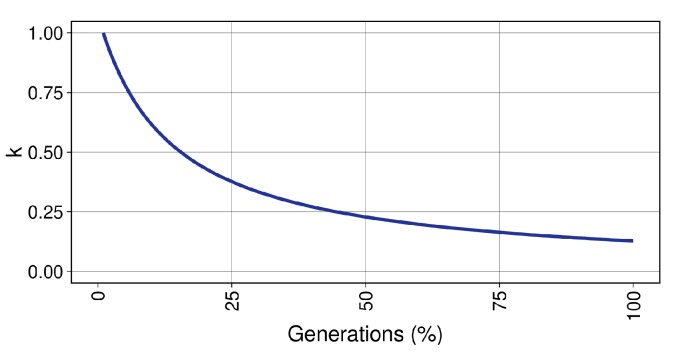
Evolution of the values of *k* during the execution of the EA.

First, each value of the individual $\vec{x}$ in *Q* is exposed to mutation according to the mutation probability $P_{m}$. If a component of $\vec{x}$ is selected for mutation, a new *M* value is randomly calculated according to a uniform probability distribution. Then, the value of Δ is obtained according to Equation (2) taking into account the upper and lower bounds of the parameter associated to each component of $\vec{x}$. Note that if the parameter being modified in $\vec{x}$ corresponds to an integer value, Δ is rounded before being added to the value of ${\vec{x}}^{\prime }\left[i\right]$ to preserve the correct parameter type. There also exists a reduction factor *k* (Equation (3)) which decreases exponentially the magnitude of the mutation (Figure 6), using the constant term γ (Equation (4)) so that, at the end of the optimisation process, *k* is equal to $k_{min}$ (0.10 in our study). This allows an initial exploration in the early stages of the algorithm and a later exploitation of the more promising areas of the search space. Finally, it is randomly decided (equally probable) to increase or decrease the current value of the parameter being mutated, and the new individual (now ${\vec{x}}^{\prime }$) is added to the new working population $Q^{\prime }$.

(2) $$\begin{array}{ccc} \Delta & = & \frac{UpBd(x[i])-LowBd(x[i])}{M}\times k \end{array}$$

(3) $$\begin{array}{ccc} k & = & e^{\frac{1-t}{\gamma }} \end{array}$$

(4) $$\begin{array}{ccc} \gamma & = & \frac{1-t_{max}}{\ln k_{min}} \end{array}$$

### 5.2. EA Parameter Optimisation

Usually, studies using optimisation algorithms include a parameter tuning stage with the objective of improving their performance and accuracy over the use of the default values. A manual tuning approach is frequently found in the literature but it depends on the previous experience of the researchers and their available resources. Consequently, it is frequently biased and incomplete.

**Figure 7:**
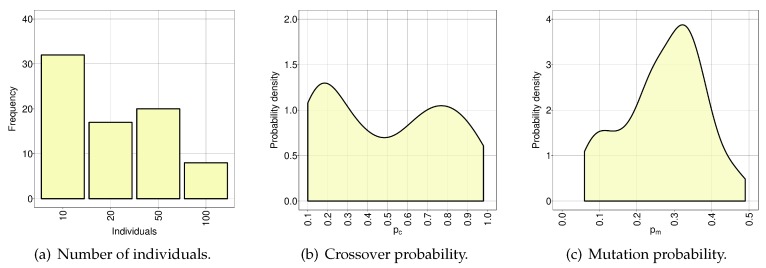
Frequency of the tests performed during the irace run for: the number of individuals (**a**); crossover probability (**b**); and mutation probability (**c**).

In the present study, we opted to use the package irace [45] as an automatic method for algorithm configuration. Irace is an implementation of iterated racing procedure which uses Friedman’s non-parametric two-way analysis of variance by ranks [46]. We defined as the irace’s variables the parameters of the EA, i.e., the number of individuals ($N_{i}$), crossover probability ($P_{c}$), and mutation probability ($P_{m}$). Then, we set the number of experiments (irace’s budget) to 500 and the confidence interval for the elimination test to 0.95. The best configurations obtained after the execution of irace are shown in Table 4 while Figure 7 shows the frequency in which each configuration of parameters was tested.

Besides the best configuration (20 individuals, $P_{c}=0.92$ and $P_{m}=0.22$), a reduced number of individuals (10), lower crossover probability and higher mutation rate were included in the top three best-rated candidates shown in Table 4. We believe that the best configuration performed better due to the fact that 20 individuals improved the sampling of the search space while avoiding a premature convergence of the EA. We also believe that 50 and 100 individuals required so many evaluations per generation (fixed number for all the configurations) that the EA was unable to progress in the search of solutions. Hence, the best configuration seems to be the most balanced solution between execution time and number of evaluations per generation.

The rest of parameters of EA are the maximum number of evaluations (100,000) and the analysis time (600 s). The former was restricted by the long evaluation times and the parallel simulation of 30 scenarios and the latter to take into account the average autonomy of UAVs.

The whole parameterisation was performed in parallel using 28 computing cores. The whole process took about 5.8 days of computing time. Note that, since the EA is a stochastic algorithm, irace had to perform several runs to evaluate each configuration with statistical confidence. The total number of performed runs was 500 and 76 different configurations were tested.

## 6. Experimental Results

Having performed the parameterisation of our EA, we used this algorithm to optimise ABISS to maximise area coverage and foster inter-swarm collaborations. We conducted a sanity check comparing our optimisation results against random search and, after that, an analysis of the results achieved were carried out taking into account other mobility models.

### 6.1. Random Search (RS)

We present in this section the Random Search (RS) algorithm to be compared with our EA as the chosen optimisation algorithm in terms of precision and efficiency.

We can see in Algorithm 5 the pseudocode of RS. This algorithm generates new configurations to be simulated and evaluated and keeps the best one found along the iterations. Depending on the case study, the solution vector would be composed of four or eight parameters.
**Algorithm 5** Pseudocode of Random Search (RS). **procedure**
RS  best←Initialisation()  **while**
notTerminationCondition()
**do**   next←getRandomConfiguration() ▹ 4 or 8 parameters depending on the case study   **if**
F(next)>F(best)
**then**    best←next            ▹ The best configuration found so far, is updated   **end if**  **end while** **end procedure**

First, an initial configuration is calculated and stored as the *best* configuration found. Then, using the same *TerminationCondition* as in EA (100,000 evaluations), new random configurations are generated, evaluated and compared against the best one found at that moment. If the new configuration happens to be better (greater fitness), it replaces the current best one (is assigned to best). This process continues until reaching the maximum number of evaluations (*TerminationCondition*). The results obtained using RS are discussed in the next section where they are also compared with EA.

### 6.2. Coverage Optimisation

The optimisation process consisted of 30 runs of the EA and RS on each case study (720 runs and 100,000 evaluations per run evaluating 30 scenarios simultaneously). The experiments were run in our simulation environment, and, at the end of the simulation, the fitness value for the configuration being evaluated was calculated, as explained in Section 4.1.

**Figure 8:**
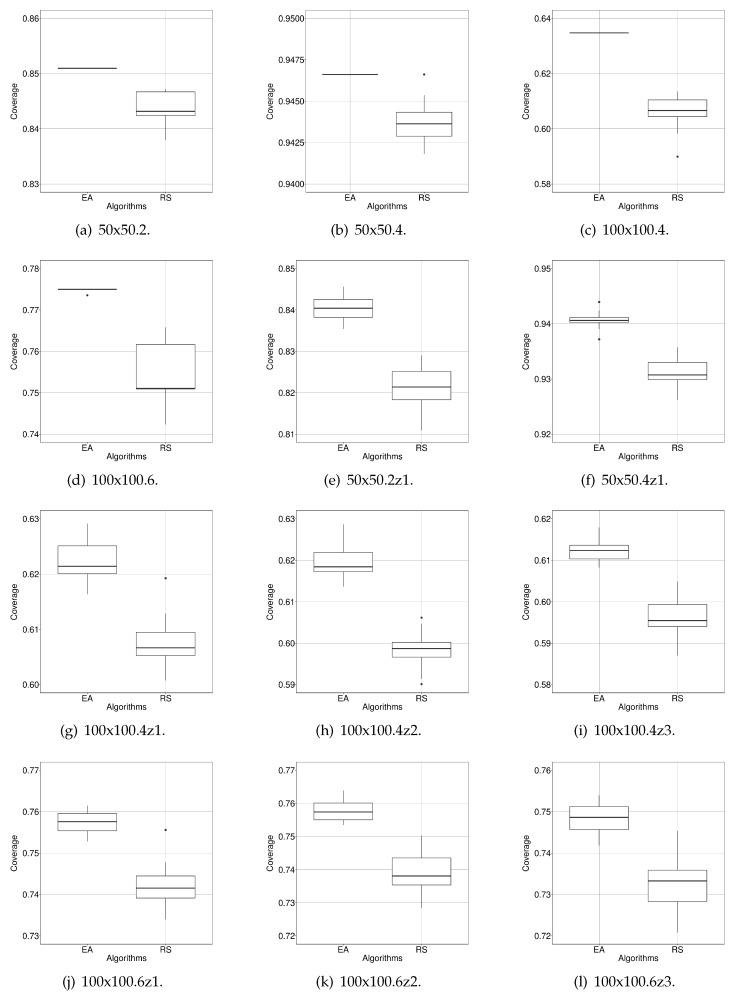
Box plots showing the distribution of values (30 runs) of EA and RS after optimising the proposed 12 case studies. As we are maximising area coverage, the higher the better.

The results obtained are shown in Table 5 where it can be seen that EA has achieved the best coverage values in all the case studies and also presented the highest accuracy. RS has found the same maximum coverage value as EA in 50x50.4. However, this RS’s result is one outlier in the distribution of results that has been found by chance. This is also supported by the last column of Table 5, which contains the results of the Wilcoxon’s tests. It shows that the comparison’s results are statistically significant (*p*-value below 0.001). Moreover, in Figure 8, the distribution of the results of RS and our EA are shown. They visually confirm that the range of the values of EA corresponds to a higher area coverage than RS.

It may seem that the improvement of EA against RS (2.8% maximum) does not justify using a complex evolutionary algorithm for addressing our problem. However, we believe that, if we take into account that 2.8% represents 280 1 × 1-m${}^{2}$ cells, the extra area covered justifies the effort. Additionally, EA turn out to be more precise than RS (smaller StDev), having converged to the same minimal value (probably the minimum) in the three first case studies.

All the experiments were performed doing parallel runs in the HPC facilities of the University of Luxembourg [47]. The total experimentation time was equivalent to 15,709 h (about 655 days).

### 6.3. Testing the Best Configuration

Having calculated the best combination of parameters for each case study, we tested it individually on each one of the 30 scenarios to obtain the results shown in Table 6. In addition to the parameter values, the total coverage obtained is also reported (mean, standard deviation and the best scenario). Note that the first four scenarios do not have unreachable zones. Consequently, no collaborations are required and the four last parameters are not needed by the mobility model.

We can observe that the average coverage is always above 60% (we analysed only the first 600 s) and that the more vehicles, the better, as expected. The small scenario (50 × 50) was explored up to 95.7%, while the big one (100 × 100) obtained a maximum 83.1% of coverage when using six vehicles (four UAVs and two UGVs).

Regarding the value of the parameters, pheromone decay ($\tau_{d}$) has always been equal to the minimum value (0.01, i.e., one unit per second) which shows the importance of the pheromone trails, and also the fact that we are not using recent coverage information in the fitness function. The pheromone radius ($\tau_{r}$) has taken always the maximum value (2) except for the last case study, confirming the importance of the vehicles’ trails, while the scan angle ($\tau_{a}$) and depth ($\tau_{m}$) have not shown a clear pattern of behaviour.

When collaboration was possible, its probability ($\pi_{c}$) has usually been kept low, except in 100x100.4z3 where it is 0.55. This is an interesting result showing that this strategy was not always preferred every time an attractor was detected. Another evidence of that is the number of collaborations which has been also regulated by the attractor scan angle ($\alpha_{a}$) and depth ($\alpha_{m}$), although these do not have a big influence in the system, as shown by the parameter sensitivity study,

Evidence shows that vehicles decided to assist each other just what was strictly necessary to cover the corresponding UZs, since collaborating implies changing their original trajectory and heading to a detected attractor. In fact, in 100x100.6z3, despite having the same layout as 100x100.4z3, inter-swarm collaboration was not chosen as a good strategy by vehicles ($\pi_{c}=0.0$).

### 6.4. ABISS vs. CACOC

Finally, we present in Table 7 a comparison between the system using only CACOC and when using ABISS, in the eight case studies having UZs. We report the total coverage and also the specific coverage of the UZs for each model. The UZ coverage was calculated taking into account only the cells included in the UZ, while for the total coverage, all the cells were used. Additionally, we include the coverage values for CACOC_0_, which is the initial mobility model presented in [29], not using the parameterisation proposed in our study. CACOC_0_ uses fixed parameters by design, i.e., $\tau_{d}=1.0$, $\tau_{r}=0$, $\tau_{a}=0$ and $\tau_{m}=2$.

It can be seen that the parameterised CACOC always outperformed CACOC_0_ since in the former the vehicles were customised to each case study. Moreover, ABISS improved both coverage metrics (Total area and UZ) for almost all case studies. In 100x100.6z3, as mentioned above, the best result is achieved when not using collaboration between vehicles. We include the second best configuration, marked by asterisks, to show that the total coverage is only 0.1% lower when using collaborations. Note that this is not the best solution found by EA, hence the asterisks.

We have to remember here that the evaluation function takes into account only the total covered area without including the percentage of the UZs covered during the optimisation process. Consequently, our results show that the evolving configurations (population of individuals in the EA) have converged to solutions in which collaboration emerged as an extra reward for the swarm members trying to cover as much of the area as possible.

## 7. Conclusions

In this article, we propose a new mobility system for inter-swarm collaboration, called Attractor Based Inter-Swarm collaborationS (ABISS), to maximise the area covered by unmanned autonomous vehicles improving the surveillance of restricted zones. Vehicles in our system follow unpredictable trajectories based on chaotic solutions of dynamic systems, pheromone trails, and use attractors to request for collaborations where they have found an unreachable zone. We propose an evolutionary algorithm adapted to this problem and used it to optimise the ABISS’ parameters in 12 case studies (30 scenarios each) comprising different map sizes, number and type of unmanned vehicles, and layouts. As a result, we obtained different configurations, which improved the coverage of the surveillance area, promoting collaboration between swarms.

We observed in most of our scenarios an emerging collaboration between vehicles, e.g., UAVs exploring water surface where a UGV was unable to access. The evolution of the parameters of the system promoted this altruistic collaboration, in which a vehicle abandons its original trajectory to explore such an unreachable area. ABISS improved the coverage of entire areas by 5.3% on average (up to 11.1%), and the exploration of unreachable zones by 13.9% (up to 40.3%), with respect to CACOC (Chaotic Ant Colony Optimisation for Coverage). In one case study, the strategy chosen by vehicles did not lead to collaborations, showing that our algorithm is able to find the best solution even in these rare scenarios. This negligible difference in the total coverage of this case study (0.1%) is related to the location of the unreachable zones and the initial trajectories of the vehicles. If we compare ABISS with the basic CACOC_0_, which cannot be parameterised, the improvements in the coverage are even higher (17.6% on average and 65.8% in UZs).

Using ABISS as a real world application for surveillance will include an offline setup stage of about 48 h. It will consist of the optimisation of the vehicles’ parameters according to the characteristics of the surveillance area and the composition of the swarms, performed by EA. After this, further EA runs will not be necessary provided the area and the swarms’ components do not change. The use of different initial conditions for the chaotic attractor will provide diverse vehicles’ trajectories which are unpredictable for intruders but deterministic for the Ground Control Station.

As a matter of future work, we plan to evaluate our proposal on other scenarios, including also other types of vehicles such as Unmanned Maritime Vehicles (UMV), and also increase the realism of our approach adding a realistic communication layer to exchange data related to pheromones and attractors. Finally, we are also interested in testing this mobility system using real autonomous vehicles (quadcopters and rovers) having already taken some steps in that direction testing the proposed trajectories using actual UAVs as well as collaborating with the SIMMS project (SIMMS: Swarm Intelligent Mission systeMS—https://simms.gforge.uni.lu/).

## Figures and Tables

**Table 1:** Pheromone action table.

Probability of Action:	Left	Ahead	Right
	$P_{L}=\frac{total-left}{2\times total}$	$P_{A}=\frac{total-ahead}{2\times total}$	$P_{R}=\frac{total-right}{2\times total}$

**Table 2:** Parameters of ABISS to be optimised.

Parameter	Symbol	Type	Units	Range
**Intra-Swarm**				
Pheromone decay	$\tau_{d}$	real	%	[0.01–0.20]
Pheromone radius	$\tau_{r}$	integer	cells	[0–2]
Pheromone scan angle	$\tau_{a}$	real	radians	[0.00–$\frac{\pi }{4}]$
Pheromone scan depth	$\tau_{m}$	integer	cells	[1–10]
**Inter-Swarm**				
Collaboration probability	$\pi_{c}$	real	–	[0.00–1.00]
Attractor scan angle	$\alpha_{a}$	real	radians	[0.00–$\frac{\pi }{4}]$
Attractor scan depth	$\alpha_{m}$	integer	cells	[1–20]
Pheromone in unreachable area	$\tau_{u}$	real	%	[0.00–1.00]

**Table 3:** Characteristics of the 12 case studies (four without UZs and eight having two UZs).

Case Study	Size	# UZ	# UAV	# UGV
50x50.2	50 × 50	0	1	1
50x50.4	50 × 50	0	2	2
100x100.4	100 × 100	0	2	2
100x100.6	100 × 100	0	4	2
50x50.2z1	50 × 50	2 (15 × 15)	1	1
50x50.4z1	50 × 50	2 (15 × 15)	2	2
100x100.4z1	100 × 100	2 (30 × 30)	2	2
100x100.4z2	100 × 100	2 (30 × 30)	2	2
100x100.4z3	100 × 100	2 (30 × 30)	2	2
100x100.6z1	100 × 100	2 (30 × 30)	4	2
100x100.6z2	100 × 100	2 (30 × 30)	4	2
100x100.6z3	100 × 100	2 (30 × 30)	4	2

**Table 4:** The three best parameter configurations of EA. The configuration chosen is in bold.

Ranking	# Individuals	*P_c_*	*P_m_*	Mean Fitness
**1st**	**20**	**0.92**	**0.22**	**0.8020**
2nd	10	0.10	0.37	0.7994
3rd	10	0.11	0.36	0.7984

**Table 5:** Results of the optimisation process performed by EA and RS and the Wilcoxon *p*-value of each statistical test. We report the fitness values obtained from 30 runs of each algorithm on the 12 case studies (four without UZs and eight having two UZs) comprising 30 instances (scenarios) each (720 optimisation runs in total). Best fitness values are in bold.

Case Study	Fitness Values (30 runs)						Wilcoxon *p*-Value
	RS			EA			
	Mean	StDev	Max	Mean	StDev	Max	
50x50.2	0.844	2.80 × 10${}^{-3}$	0.847	**0.851**	**0.00 × 10${}^{0}$**	**0.851**	0.000
50x50.4	0.944	1.43 × 10${}^{-3}$	**0.947**	**0.947**	**0.00 × 10${}^{0}$**	**0.947**	0.000
100x100.4	0.607	5.24 × 10${}^{-3}$	0.613	**0.635**	**0.00 × 10${}^{0}$**	**0.635**	0.000
100x100.6	0.755	7.08 × 10${}^{-3}$	0.766	**0.775**	**2.64 × 10${}^{-4}$**	**0.775**	0.000
50x50.2z1	0.822	5.01 × 10${}^{-3}$	0.829	**0.840**	**2.80 × 10${}^{-3}$**	**0.846**	0.000
50x50.4z1	0.931	2.41 × 10${}^{-3}$	0.936	**0.941**	**1.14 × 10${}^{-3}$**	**0.944**	0.000
100x100.4z1	0.607	3.65 × 10${}^{-3}$	0.619	**0.622**	**3.12 × 10${}^{-3}$**	**0.629**	0.000
100x100.4z2	0.599	3.69 × 10${}^{-3}$	0.606	**0.619**	**3.54 × 10${}^{-3}$**	**0.629**	0.000
100x100.4z3	0.596	4.09 × 10${}^{-3}$	0.605	**0.612**	**2.22 × 10${}^{-3}$**	**0.618**	0.000
100x100.6z1	0.742	4.43 × 10${}^{-3}$	0.756	**0.757**	**2.55 × 10${}^{-3}$**	**0.761**	0.000
100x100.6z2	0.739	5.87 × 10${}^{-3}$	0.750	**0.758**	**2.95 × 10${}^{-3}$**	**0.764**	0.000
100x100.6z3	0.732	5.41 × 10${}^{-3}$	0.745	**0.748**	**3.40 × 10${}^{-3}$**	**0.754**	0.000

**Table 6:** Best configuration for each case study (parameters) followed by the mean, standard deviation and best coverage obtained when testing them in the 30 corresponding scenarios.

Case Study	$\tau_{d}$	$\tau_{r}$	$\tau_{a}$	$\tau_{m}$	$\pi_{c}$	$\alpha_{a}$	$\alpha_{m}$	$\tau_{u}$	Coverage (%)		
									Mean	StDev	Best
50x50.2	0.01	2	0.26	5	–	–	–	–	85.1	2.8	90.3
50x50.4	0.01	2	0.23	4	–	–	–	–	94.7	0.8	95.7
100x100.4	0.01	2	0.22	8	–	–	–	–	63.5	3.1	69.2
100x100.6	0.01	2	0.38	7	–	–	–	–	77.5	2.9	82.0
50x50.2z1	0.01	2	0.36	4	0.21	0.08	18	0.59	84.6	4.2	89.8
50x50.4z1	0.01	2	0.15	4	0.35	0.06	18	0.67	94.4	1.0	95.6
100x100.4z1	0.01	2	0.15	10	0.22	0.19	7	0.88	62.9	2.6	67.8
100x100.4z2	0.01	2	0.34	8	0.24	0.03	7	0.76	62.9	2.7	67.1
100x100.4z3	0.01	2	0.32	6	0.55	0.17	11	0.75	61.8	3.4	67.4
100x100.6z1	0.01	2	0.35	6	0.35	0.28	7	0.26	76.1	3.2	80.7
100x100.6z2	0.01	2	0.35	7	0.33	0.36	3	0.79	76.4	2.7	83.1
100x100.6z3	0.01	1	0.37	7	0.00	0.06	16	0.82	75.4	2.6	80.6

**Table 7:** Comparison between the coverage values obtained by the non-collaborative approaches (CACOC_0_ and CACOC) and ABISS in case studies having unreachable zones (mean of 30 scenarios). The improvements achieved by ABISS with respect to CACOC_0_ and CACOC are also reported. The second best configuration values are indicated by asterisks. The best results are in bold.

Case Study	Coverages (%)									
	CACOC_0_		CACOC		ABISS					
	Total	UZ	Total	UZ	Total	Improvement vs.		UZ	Improvement vs.	
						CACOC_0_	CACOC		CACOC_0_	CACOC
50x50.2z1	76.4	50.5	80.4	58.1	**84.6**	+10.7%	+ 5.2%	**70.1**	+38.8%	+ 20.7%
50x50.4z1	89.9	70.9	93.0	83.9	**94.4**	+ 5.0%	+ 1.5%	**88.8**	+25.2%	+5.8%
100x100.4z1	51.2	12.9	59.8	36.6	**62.9**	+ 22.9%	+ 5.2%	**39.6**	+ 207.0%	+ 8.2%
100x100.4z2	47.8	38.3	56.6	57.2	**62.9**	+ 31.6%	+ 11.1%	**67.1**	+ 75.2%	+ 17.3%
100x100.4z3	50.4	24.6	57.2	36.7	**61.8**	+ 22.6%	+ 8.0%	**51.5**	+ 109.3%	+ 40.3%
100x100.6z1	64.0	25.7	71.9	41.0	**76.1**	+ 18.9%	+ 5.8%	**55.4**	+ 115.6%	+ 35.1%
100x100.6z2	62.2	48.5	70.1	71.8	**76.4**	+ 22.8%	+ 9.0%	**77.3**	+ 59.4%	+ 7.7%
100x100.6z3	63.7	39.6	**75.4**	**67.4**	* 75.3	+ 18.2%	−0.1%	* 65.8	+ 66.2%	−2.4%
**Mean:**	63.2	38.9	70.6	56.6	**74.3**	17.6%	5.3%	**64.5**	65.8%	13.9%
